# Does Guideline‐Directed Medical Therapy Reduce the Incidence of Ventricular Arrhythmias in Patients With HFrEF?

**DOI:** 10.1002/joa3.70253

**Published:** 2025-12-08

**Authors:** Yu Nomoto, Naoya Kataoka, Teruhiko Imamura

**Affiliations:** ^1^ Second Department of Internal Medicine University of Toyama Toyama Japan

**Keywords:** guideline‐directed medical therapy, heart failure, ventricular tachycardia

To editor,

In the present study, among 139 patients with heart failure with reduced ejection fraction (HFrEF) who had a dual chamber implantable cardioverter‐defibrillator (ICD) or cardiac resynchronization therapy‐defibrillator (CRT‐D), twelve patients (8.6%) experienced sustained ventricular tachycardia (VT). Of these, seven (58%) were non‐compliant with heart failure guideline‐directed medical therapy (GDMT) [1]. This finding suggests a strong correlation between poor adherence to GDMT and the occurrence of VT. Recent reports indicate that the proportion of sudden cardiac death among HFrEF patients has been decreasing, likely due to the advent of novel heart failure medications and the widespread implementation of GDMT [2]. From the perspective of GDMT, we believe several points in this study warrant further discussion.

GDMT was defined as the use of both beta‐blockers and a renin–angiotensin system inhibitor (RASi)—including an angiotensin‐converting enzyme inhibitor, an angiotensin receptor blocker, or an angiotensin receptor‐neprilysin inhibitor—while mineralocorticoid receptor antagonists (MRAs) and sodium–glucose cotransporter 2 (SGLT2) inhibitors were not included. According to current heart failure guidelines, GDMT now encompasses all four drug classes, including MRAs and SGLT2 inhibitors. Therefore, the definition of GDMT in this study does not fully align with contemporary standards.

The study categorized patients simply based on whether GDMT was administered or not, without considering dose optimization. However, the up‐titration of GDMT agents to target or maximally tolerated doses is a key component of effective therapy. Previous studies have shown that patients with HFrEF who achieved up‐titration of RASi, beta‐blockers, and MRAs had significantly lower mortality rates [3]. While the prescription rate was high in the present study, it remains unclear whether adequate dose escalation was achieved.

Beyond the GDMT perspective, another interesting finding is the high incidence of nonsustained VT, despite the relatively young cohort. Compared with prior studies, this may be due to differences in the underlying etiology. In older populations, valvular heart disease and coronary artery disease are more prevalent, whereas cardiomyopathies may have been overrepresented in this cohort. Therefore, a detailed analysis of the underlying cardiac pathology in these patients would be essential for further interpretation.

## Funding

The authors have nothing to report.

## Ethics Statement

The authors have nothing to report.

## Consent

The authors have nothing to report.

## Conflicts of Interest

The authors declare no conflicts of interest.

## Data Availability

The manuscript does not include any original data.
